# Pharmacokinetic modeling of [^11^C]flumazenil kinetics in the rat brain

**DOI:** 10.1186/s13550-017-0265-4

**Published:** 2017-02-22

**Authors:** Isadora Lopes Alves, David Vállez García, Andrea Parente, Janine Doorduin, Rudi Dierckx, Ana Maria Marques da Silva, Michel Koole, Antoon Willemsen, Ronald Boellaard

**Affiliations:** 1 Department of Nuclear Medicine and Molecular Imaging, University Medical Center Groningen, University of Groningen, Groningen, The Netherlands; 2 Laboratory of Medical Imaging, School of Physics, Pontifícia Universidade Católica do Rio Grande do Sul, Porto Alegre, Brazil; 3 Department of Nuclear Medicine and Molecular Imaging, KU Leuven, Leuven, Belgium

**Keywords:** [^11^C]Flumazenil, Pharmacokinetic modeling, Rat, GABA_A_ receptors, PET

## Abstract

**Background:**

Preferred models for the pharmacokinetic analysis of [^11^C]flumazenil human studies have been previously established. However, direct translation of these models and settings to animal studies might be sub-optimal. Therefore, this study evaluates pharmacokinetic models for the quantification of [^11^C]flumazenil binding in the rat brain.

Dynamic (60 min) [^11^C]flumazenil brain PET scans were performed in two groups of male Wistar rats (tracer dose (TD), *n* = 10 and pre-saturated (PS), *n* = 2). Time-activity curves from five regions were analyzed, including the pons (pseudo-reference region). Distribution volume (V_T_) was calculated using one- and two-tissue compartment models (1TCM and 2TCM) and spectral analysis (SA). Binding potential (BP_ND_) was determined from full and simplified reference tissue models with one or two compartments for the reference tissue (FRTM, SRTM, and SRTM-2C). Model preference was determined by Akaike information criterion (AIC), while parameter agreement was assessed by linear regression, repeated measurements ANOVA and Bland-Altman plots.

**Results:**

1TCM and 2TCM fits of regions with high specific binding showed similar AIC, a preference for the 1TCM, and good V_T_ agreement (0.1% difference). In contrast, the 2TCM was markedly preferred and necessary for fitting low specific-binding regions, where a worse V_T_ agreement (17.6% difference) and significant V_T_ differences between the models (*p* < 0.005) were seen. The PS group displayed results similar to those of low specific-binding regions. All reference models (FRTM, SRTM, and SRTM-2C) resulted in at least 13% underestimation of BP_ND_.

**Conclusions:**

Although the 1TCM was sufficient for the quantification of high specific-binding regions, the 2TCM was found to be the most adequate for the quantification of [^11^C]flumazenil in the rat brain based on (1) higher fit quality, (2) lower AIC values, and (3) ability to provide reliable fits for all regions. Reference models resulted in negatively biased BP_ND_ and were affected by specific binding in the pons of the rat.

## Background

[^11^C]Flumazenil is a well-established and widely used radiotracer in positron emission tomography (PET) studies in humans [1] as a GABA_A_ antagonist. It binds to the benzodiazepine binding site of the GABA_A_ receptor, allowing PET imaging of [^11^C]flumazenil uptake to enable the in vivo visualization and quantification of processes related to neuronal loss and integrity [2, 3]. More specifically, flumazenil displays comparable levels of binding to the GABA_A_ receptors containing α1, α2, α3, or α5 subunits, which is why this compound is considered to be not subtype-selective [4]. For these reasons, [^11^C]flumazenil can be considered a versatile PET tracer and it has been used in the study and assessment of several conditions, such as neuronal damage in head injury [5], epilepsy [6], stroke-induced penumbral areas of infarction [7], and Alzheimer’s disease [8].

The quantification of [^11^C]flumazenil uptake and binding is often performed by pharmacokinetic modeling, with the aid of compartmental models [9]. For the application of those models, information on tracer delivery is needed. This information can be obtained either in a direct form, by arterial sampling and the construction of a plasma input function, or in an indirect manner making use of a reference tissue devoid of receptors of interest [10]. Previous human studies [11, 12] evaluated the performance of some of these models (plasma input and reference tissue based) and established which ones provided a more robust and reliable quantification of [^11^C]flumazenil receptor binding. For plasma input methods, the one tissue compartment model (1TCM) was determined to be the preferred model [11]. Among the reference-based models for [^11^C]flumazenil quantification, the simplified reference tissue model (SRTM) was considered valid and robust [13], with the use of the pons as the reference tissue, based on the fact that this region shows a low density of GABA_A_ receptors [14].

However, the superiority, validity, and accuracy of the 1TCM and the SRTM for the quantification of [^11^C]flumazenil binding have only been assessed in human studies and, therefore, are related to the behavior of this radiotracer in the human brain. Surprisingly, there has not yet been a careful assessment of the performance of the different pharmacokinetic models for rat studies. Nonetheless, pharmacokinetic modeling can be of particular relevance in the pre-clinical setting, since animal studies are often applied in drug development, longitudinal disease monitoring, or treatment response assessment. There, assessing the full tracer kinetic profile in tissue is preferable above semi-quantitative measures such as standard uptake values (SUVs) or SUV ratios (SUVR) [15, 16]. Since anatomical and physiological variations between species can affect tracer kinetics, the suitability of the clinically appropriate kinetic models for [^11^C]flumazenil brain kinetics can be questioned. Therefore, the purpose of this study was to investigate which pharmacokinetic model is most appropriate for the quantification of [^11^C]flumazenil binding in pre-clinical rat studies. In order to do so, we retrospectively analyzed PET rat brain data, as well as simulated data, by a number of different methods, including compartmental (plasma input and reference tissue based) and non-compartmental methods.

## Methods

Data from male outbred Wistar-Unilever rats (*n* = 12) obtained from Harlan (Horst, The Netherlands) and part of a pre-clinical study were analyzed. The pre-clinical study, of which the results will be reported elsewhere, divided the rats into three groups: controls (CRTL, *n* = 5), rats displaying neuroinflammation as a result of infection with the herpes encephalitis virus (HSV, *n* = 5) [17–19], and rats pre-saturated with cold flumazenil (PS, *n* = 2). For the purpose of this pharmacokinetic modeling study, rats from the CRTL and the HSV group were combined into one group which received a tracer dose only (TD, *n* = 10). The PS group remained unchanged and as a separate group for additional analysis (Table 1). Although outside the scope of this study, the CTRL and HSV groups were tested for differences in plasma and metabolite curves, as well as for regional quantitative endpoint parameters derived from each tested pharmacokinetic models and no differences were found between the two groups, supporting the construction of the TD group.

**Table 1:** Overview of the animal groups, injected activity, and mass. Data are expressed as mean ± SD

	Injected activity	Injected mass	Animal weight
Group	(MBq)	(nmol)	(kg)
Tracer dose (*n* = 10)	59.3 ± 22.6	3.3 ± 2.4	0.273 ± 0.03
Pre-saturated (*n* = 2)	71.9 and 59.1	1.9 and 5.6	0.300 and 0.295

All animal experiments were performed according to the Dutch Law on Animal Experiments and approved by the Institutional Animal Care and Use Committee of the University of Groningen (6264B).

### PET imaging

Animals were anesthetized with 5% isoflurane (maintained at 1.5–2.0%) mixed with medical air with 1.5–2 ml/min flow. Next, animals were positioned in the PET camera (Focus 220, Siemens Medical Solutions, USA) with the head in the field of view and in trans-axial position. After a point-source (Co-57) transmission scan, an automatic pump was used for the bolus injection of [^11^C]flumazenil over 60s. Dynamic PET data were acquired in list mode for 60 min, starting from the moment the tracer entered the body. For the PS group, unlabeled flumazenil (330 nmol in 200 μl of 15% ethanol in saline) was injected intravenously 5 min before tracer administration [20].

The precursor of [^11^C]flumazenil and the cold flumazenil were obtained from ABX (art. 1700 and 1710, respectively) in order to ensure high chemical purity (>95%). The labeled [^11^C]flumazenil was then synthesized in our laboratory at the UMCG as described elsewhere [21]. Moreover, the cold flumazenil compound was used as standard for the HPLC settings for both the radiotracer synthesis and for the metabolite analysis. It was also the compound used for the pre-saturation of the PS group.

### Image processing

List-mode data was reconstructed into 21 frames (6 × 10, 4 × 30, 2 × 60, 1 × 120, 1 × 180, 4 × 300 and 3 × 600 s) including all necessary corrections to obtain quantitative emission images. Sinograms were Fourier rebinned and reconstructed by a 2D-OSEM algorithm [22] with 4 iterations and 16 subsets, resulting in 128 × 128 × 95 matrix images, with 0.63 mm pixel width and 0.79 mm slice thickness. All subsequent PET image analysis, including pharmacokinetic modeling, was performed with PMOD v3.7 (PMOD Technologies Ltd., Switzerland).

Individual dynamic images were automatically coregistered to a [^11^C]flumazenil template [23] pre-aligned to the Paxinos stereotaxic coordinates system. Bilateral volumes of interest (VOIs) were extracted from a set of previously constructed regions [23], including the whole brain and regions with high GABA_A_ expression (the frontal cortex and the hippocampus), and low GABA_A_ expression (cerebellum, medulla, and pons). Due to their small size and potential vulnerability to spillover effects, the VOIs from medulla and pons were adapted and reduced in order to further separate them from neighboring high tracer uptake regions. Finally, time-activity curves (TACs) were generated by projecting the pre-defined VOIs onto the dynamic images.

### Determination of individual metabolite corrected plasma input curves

For the determination of a metabolite corrected plasma input function, arterial blood samples of 0.1 ml were acquired (*n* = 16) and for 2–3 of the time points, larger samples (0.6 ml) were collected for metabolite analysis. These 2-3 time points were collected at 5, 10, 15, 30, 45 or 60 min post injection, in order to adequately sample the metabolites dynamics. Equivalent volumes of saline were injected to avoid a decrease in blood pressure. For each sample, blood and plasma were separated and measured in a gamma counter (LKB-Wallac, Finland) for the construction of both blood and plasma input curves.

The metabolites were determined by first diluting the plasma with an equivalent volume of acetonitrile and mixing it with a vortex for 1 min. Next, it was centrifuged at 5.300 rpm for 3 min. The supernatant was then filtered through a Millipore filter (Millex-HV 4 mm syringe filter, pore 0.45 μm) and an equivalent volume of water was added, and the volume adjusted to 1 ml with an HPLC mobile phase. The resulting solution was analyzed by HPLC using an Alltima RP-C18 column (5 μm, 10 × 250 mm) and a mobile phase of acetonitrile/1 mM H3PO4 (25/75) at a flow of 5 ml/min.

Next, a population metabolite curve was constructed by averaging the metabolite samples of the individual rats and fitting the average curve with a single-exponential function. Finally, the population average metabolite curve was used for the metabolite correction of the individual plasma input functions.

### Pharmacokinetic modeling

Plasma input models included the one- and two-tissue compartment models (1TCM and 2TCM, respectively), and the spectral analysis (SA) [24]. The SA method was applied with 50 basis functions, the minimum exponential coefficient set to 0.001 s^−1^, and the maximum exponential coefficient to 1 s^−1^. The range of exponential coefficients was determined based on (1) the range of V_T_ obtained from 1TCM and 2TCM and (2) the suggested limits of upper and lower bounds as described previously [25, 26]. The number of grid components (50) was chosen in order to balance good coverage of basis functions and computational efficiency. Model fits were performed with individual metabolite corrected plasma curves as input functions and a fixed blood volume fraction of 5% of the whole blood curve. Blood delay was calculated for the whole brain and subsequently fixed for the remaining regions. The distribution volume (V_T_) was the parameter of interest for all plasma input models.

Following previous studies, the pons was considered as the reference region and its TAC served as input function for the reference-based models. The full reference tissue model (FRTM) and two versions of the simplified reference tissue model, with either one (SRTM[27]) or two (SRTM-2C[10]) compartments for the reference region, were applied to the data. The parameter of interest for the reference tissue-based models was the non-displaceable binding potential (BP_ND_).

Finally, BP_ND_ was also indirectly determined from the V_T_ estimates of the plasma input models (BP_ND_ = (V_T_/V_T_ reference)–1 = DVR–1) [28] for method comparison.

### Simulations

At first, a set of noiseless TACs (*n* = 10) was generated to assess whether model preference was associated to the level of specific binding relative to the non-specific signal present in that particular region. For that purpose, a three-tissue compartment model (3TCM) with varying levels of specific binding was used for the generation of TACs, such that these closely matched the shapes and amplitudes of those seen in the pre-clinical data. First, an average plasma curve was determined from the animal data and used as input function. Next, representative rate constants from the 2TCM fits were determined and set as starting point for the generation of TACs. K_1_, k_2_, and k_4_ were set to the average values of the TD group parameters. Assuming complete receptor blocking in the PS group, k_5_ and k_6_ were defined as the average values of k_3_ and k_4_ of that group. Next, the different binding levels were generated by varying k_3_ from 0.2 to 2.6 min^−1^. An overview of all micro and macro parameters used for the simulated TACs can be found in Table 2. Next, 100 TACs per k_3_ were generated by adding Poisson-like noise to the noiseless TACs, with zero mean and a standard deviation corresponding to 5 and 10% of the average uptake value of the last two frames.

**Table 2:** Overview of the micro and macro parameters used for the simulated TACs

Micro parameters	Macro parameters		
k_3_	V_T_	DVR–1	BP_ND_
(min^−1^)	(K_1_/k_2_) × [1 + (k_3_/k_4_) + (k_5_/k_6_)]		(k_3_/k_4_)
0.20	1.44	0	1.17
0.35	1.75	0.22	2.05
0.50	2.06	0.43	2.94
0.65	2.38	0.65	3.82
0.80	2.69	0.87	4.70
0.95	3.00	1.09	5.58
1.10	3.32	1.30	6.47
1.25	3.63	1.52	7.35
1.40	3.95	1.74	8.23
1.55	4.26	1.96	9.11
1.70	4.58	2.18	10.0
1.85	4.89	2.39	10.8
2.00	5.20	2.61	11.7
2.15	5.51	2.83	12.6
2.30	5.83	3.05	13.5
2.45	6.14	3.27	14.4
2.60	6.46	3.48	15.3

Fixed micro parameters (min^−1^) K_1_ = 0.8; k_2_ = 2.25; k_4_ = 0.17; k_5_ = 0.15; k_6_ = 0.08

The simulated TACs were subsequently analyzed with the same models used for the animal data. V_T_ and BP_ND_ from simulated data were compared to the theoretical values derived from the 3TCM configuration (Table 2). Model preference was assessed for the noiseless TACs, as well as for the different noise levels.

### Model comparison and statistical analysis

Statistical data analysis was performed with IBM SPSS Statistics 22 for both animal and simulated data, and the results are presented as mean ± standard deviation (SD).

Model preference between 1TCM and 2TCM was assessed for each region based on the Akaike information criterion (AIC) [29]. Regional differences in V_T_ values estimated from the three different models (1TCM, 2TCM, and SA) were tested for significance by repeated measures ANOVA and subsequent paired *t* tests. Parameter estimates were also compared between all the six models by linear regression analysis and Bland-Altman plots. All results were considered significant when *p* < 0.05 and, whenever suited, subsequently corrected for multiple comparisons by applying the Bonferroni-Holm correction.

Data from the PS group was excluded from the linear regression analysis. This was done in order to avoid two clear clusters of different V_T_ magnitudes in the linear plot, which could affect the correlation values.

## Results

**Fig. 1:**
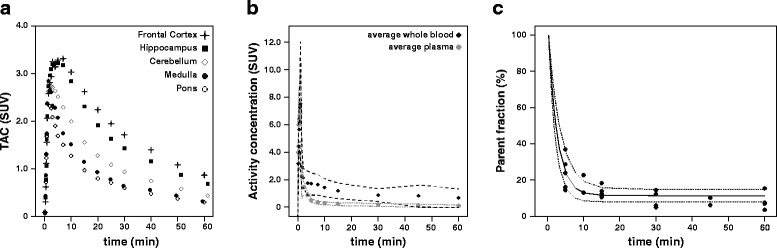
Representative TACs and plasma input function. **a** Representative TACs of the five regions analyzed in the TD group. **b** Mean whole blood and plasma input curves (*black* and *gray circles*, respectively) and the 95% confidence intervals as defined by mean ± 1.96*SD displayed in *dashed lines*. **c** Parent plasma curve in percentage, determined as 1—the average percentage of metabolites (*solid black line*), the 95% confidence intervals (mean ± 1.96*SD), displayed as *dashed lines* and the individual samples (*black circles*)

Figure 1 shows some representative time-activity curves, input functions, and metabolite data.

### Model preference

**Fig. 2:**
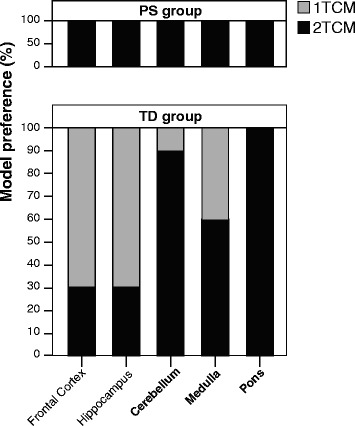
Model preference for different regions based on AIC values. Region-wise display of model preference based on AIC values for the tracer dose (TD, *n* = 10) and the pre-saturated (PS, *n* = 2) groups. High-density GABA_A_ regions (frontal cortex and hippocampus) of the TD group showed a preference for the 1TCM (*gray*), while low-density regions (cerebellum, medulla, and pons) showed a preference for the 2TCM (black). All the regions in the PS group showed a preference for the 2TCM

Model preference displayed a region-dependent behavior for the TD group (Fig. 2). A clear preference (>70%) for the 1TCM was seen in regions with high expression of GABA_A_ receptors (frontal cortex and hippocampus). However, the 2TCM was necessary to obtain satisfactory fits in regions with low GABA_A_ receptor expression such as medulla, cerebellum, and pons (60, 90, and 100% preference, respectively). Moreover, the spectra of SA analysis provided comparable information, with a prevalence of two peaks (representing two distinct compartments) for the regions of pons, medulla, and cerebellum (100, 70, and 90%). Frontal cortex showed two peaks in only two animals (20%) and hippocampus in three (30%).

**Fig. 3:**
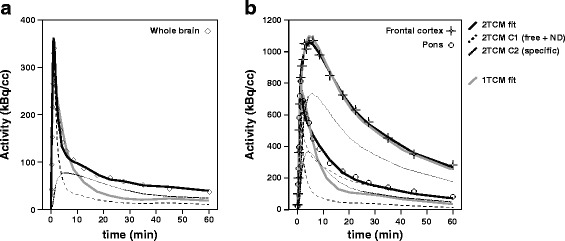
Representative 1TCM and 2TCM model fits. **a** Representative (*n* = 1) TAC and 1TCM and 2TCM model fits for the whole brain of an animal from the PS group. **b** Representative (*n* = 1) TACs and 1TCM and 2TCM model fits for the frontal cortex (*square*) and the pons (*circle*) of an animal from the TD group. The individual data points (*black dots*) and two models fits are displayed: the 1TCM (*gray solid line*) and the 2TCM (*black solid line*) with the separate compartments (free + non-displaceable, *black dashed line*; specific, *black dotted line*)

In the PS group, 2TCM fits resulted in lower AIC values for all brain regions (Fig. 2). Moreover, visual inspection showed that 1TCM fits were not satisfactory (Fig. 3a) and, generally, those resulted in an underestimation of both the peak and the tail in the TACs of the PS group. In the TD group, a similar underestimation can be seen in regions such as the pons, but not in high-binding ones such as the frontal cortex (Fig. 3b).

**Fig. 4:**
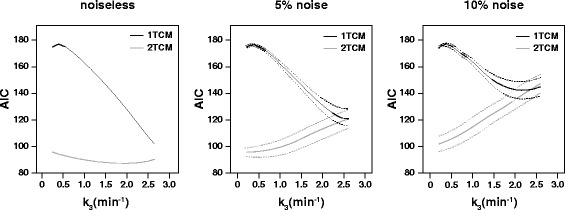
Model preference (based on AIC) of simulated TACs. A figure on model preference for simulated TACs of increasing specific binding and noise levels. On the left, a plot of AIC values for the 1TCM (*black*) and the 2TCM (*gray*) for simulated noiseless TACs with increasing levels of specific-binding (increasing k_3_, *x-axis*). The two graphs on the right represent mean ± SD (*solid line*, *dashed lines*) for the AIC values of 1TCM (*black*) and 2TCM (*gray*) fits with 5 and 10% added noise

Results from the simulated data displayed a similar pattern (Fig. 4), with AIC values varying in relation to different levels of specific binding (small k_3_). For the noiseless TACs, AIC values of 1TCM were always higher than 2TCM fits, and they decreased for increasing k_3_. When 5% noise was added, AIC for the 2TCM increased with larger k_3_ such that a small overlap in model preference was seen for the highest binding TAC. The addition of 10% noise strengthened this effect (Fig. 4), with the overlap in AIC values occurring at earlier k_3_ values.

### Distribution volume

Following the AIC results, the 2TCM V_T_ were considered as reference values for model comparison as it was the model which provided reliable fits across all brain regions.

**Fig. 5:**
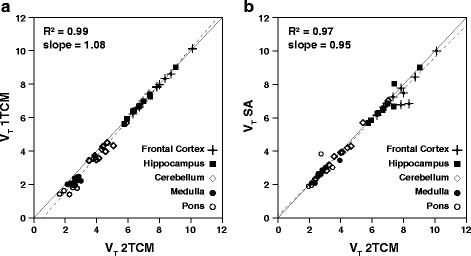
Linear regression analysis between different models. Linear regression analysis between 1TCM and 2TCM (**a**) and between SA and 2TCM (**b**). The *solid line* represents the line of identity, and the *dashed line* corresponds to the regression fit

For the TD group, a significant correlation (*p* < 0.001) was found between V_T_ from different methods for all regions (Figure 5). Moreover, a significant difference between V_T_ estimates was found for the low-binding regions, which corresponded to differences between 1TCM and 2TCM V_T_ (*p* < 0.005), as well as between 1TCM and SA V_T_ (*p* < 0.05). An overview of V_T_ values for each of the models is presented in Table 3.

**Table 3:** Regional distribution volume (V_T_) estimates from the three different methods for each of the groups. Data are expressed as mean ± SD for the tracer dose group (*n* = 10) and as individual values for the pre-saturated group (*n* = 2)

		V_T_	
Region	2TCM	1TCM	SA
Tracer dose (*n* = 10)			
Frontal cortex	7.81 ± 1.10	7.80 ± 2.38	7.15 ± 1.17
Hippocampus	6.86 ± 0.94	6.84 ± 2.08	6.55 ± 0.95
Cerebellum*	4.44 ± 0.67	4.11 ± 1.24	4.33 ± 0.65
Medulla*	2.77 ± 0.48	2.35 ± 0.71	2.66 ± 0.39
Pons*	2.54 ± 0.40	2.10 ± 0.61	2.45 ± 0.33
Pre-saturated (*n* = 2)			
Frontal cortex	1.23 and 1.10	0.67 and 0.73	1.00 and 0.89
Hippocampus	1.07 and 0.92	0.60 and 0.62	1.09 and 0.68
Cerebellum	0.98 and 0.91	0.52 and 0.58	1.00 and 0.69
Medulla	0.89 and 0.80	0.46 and 0.56	0.69 and 0.83
Pons	0.99 and 0.88	0.55 and 0.59	0.80 and 0.72

*Significant differences were found in these regions between 1TCM and 2TCM estimates (*p* < 0.005) as well as between 1TCM and SA (*p* < 0.05)

**Fig. 6:**
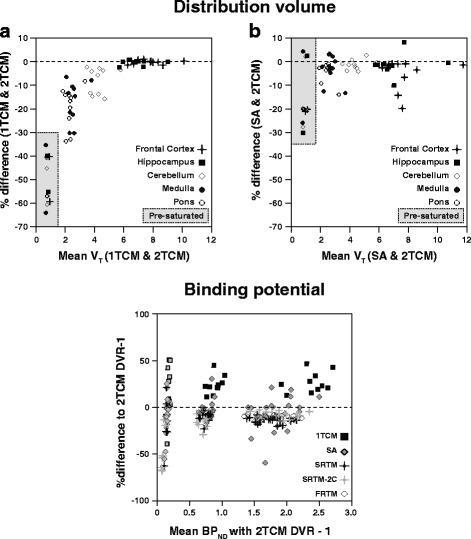
Bland-Altman plots of agreement of V_T_ and BP_ND_ estimates of different models. Bland-Altman plots for analysis of agreement in V_T_ and BP_ND_ values between different models. On the top, Bland-Altman plots for agreement between V_T_ estimates from SA and 2TCM (**a**) and from 1TCM and 2TCM (**b**). On the *bottom*, a Bland-Altman plot for agreement between BP_ND_ estimates from five different methods in comparison with DVR–1 from the 2TCM. The *dashed lines* represent zero difference and the *y-axis* is expressed in %difference. More information on the %difference and 95% limits of agreement for each method can be found in Table 4

The Bland-Altman plots also showed region-dependent differences in V_T_ estimation of the TD group, which were, percentage-wise, markedly larger for low-density regions (Fig. 6). The 1TCM V_T_ resulted in small (negative) differences compared to the 2TCM V_T_ for the frontal cortex and hippocampus (−0.20 and −0.32%, respectively), while medulla and pons showed the largest differences between the two models (−16.9 and −19.1%, respectively). Cerebellum could be considered an intermediate region, both in terms of V_T_ values (Table 3) and in differences between the models (−8.0%). The V_T_ values estimated from SA also resulted in region-dependent differences to the 2TCM, with the largest overall differences observed in the frontal cortex (−5.16%) and the smallest seen in the pons (0.44%), as can be observed in Fig. 6b. However, the variability in these differences was higher than what was seen between 1TCM and 2TCM, corresponding to wider 95% limits of agreement (Table 4).

**Table 4:** Bland-Altman analysis of V_T_, DVR–1 and BP_ND_ of animal data

	Animal data		
	% difference (95% L.A.)		
V_T_	1TCM	SA	2TCM^a^
Frontal cortex	−0.20 (−1.93 to 1.52)	−5.16 (−18.2 to 7.87)	–
Hippocampus	−0.32 (−2.50 to 1.84)	−1.43 (−10.0 to 7.15)	–
Cerebellum	−8.01 (−19.0 to 3.01)	−1.31 (−5.42 to 2.78)	–
Medulla	−16.9 (−34.0 to 0.24)	−3.54 (−14.4 to 7.32)	–
Pons	−19.1 (−35.9 to −2.37)	0.44 (−24.2 to 25.0)	–
*Overall*	−8.91 (−28.4 to 10.5)	−2.20 (−16.2 to 11.8)	–
DVR–1	1TCM	SA	
Frontal cortex	26.7 (4.74 to 48.7)	−9.01 (−53.3 to 35.3)	–
Hippocampus	28.3 (5.72 to 50.8)	−2.53 (−31.5 to 26.4)	–
Cerebellum	24.1 (4.75 to 43.2)	1.70 (−22.8 to 26.2)	–
Medulla	28.5 (−44.7 to 101)	−7.02 (−49.3 to 35.3)	–
Pons	–	–	–
*Overall*	26.9 (−12.6 to 66.4)	−4.21 (−39.8 to 31.4)	–
BP_ND_	FRTM	SRTM	SRTM-2C
Frontal cortex	−12.5 (−20.3 to −4.67)	−13.8 (−21.5 to 6.16)	−7.74 (−15.3 to −0.22)
Hippocampus	−11.0 (−17.9 to −4.05)	−12.5 (−19.6 to −5.40)	−7.59 (−12.9 to −2.24)
Cerebellum	−6.54 (−15.9 to 2.82)	−9.58 (−22.3 to 3.14)	−13.7 (−29.1 to 1.72)
Medulla	−25.6 (−152 to 101)	−27.7 (−134 to 78.8)	−66.2 (−151 to 18.8)
Pons	–	–	–
*Overall*	−13.9 (−76.7 to 48.9)	−15.9 (−69.6 to 37.8)	−23.8 (−88.0 to 40.4)

^a^The 2TCM is the model of reference for comparisons. The pons is the reference region for reference-based modeling

**Fig. 7:**
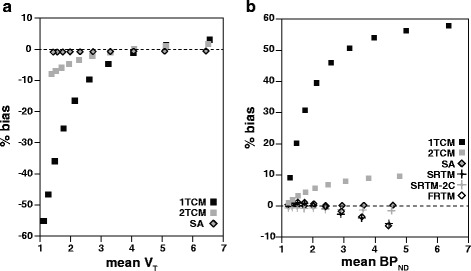
Bland-Altman plots of parameter agreement for simulated TACs. Bland-Altman plots for the analysis of parameter agreement for simulated TACs. **a** Bland-Altman plot of agreement between V_T_ estimates from 1TCM (*black square*), 2TCM (*gray square*), and SA (*gray-filled diamond*) compared to theoretical values. **b** Bland-Altman plot of agreement between DVR–1 and BP_ND_ estimates from different methods compared to theoretical values. The *dashed lines* represent zero difference and the *y-axis* is expressed in %bias

The Bland-Altman plot of the simulated data showed a bias pattern comparable to what was observed in the animal data (Fig. 7). V_T_ values from the SA method demonstrated an overall constant and small negative bias (−0.76%) compared to theoretical values. For the 1TCM and the 2TCM, the overall bias was −19.1 and −2.94%, respectively, and it was also strongly dependent on the level of binding present in the TAC (Fig. 7).

### Indirect and direct binding potential

DVR–1 from the 2TCM were considered as reference values for model comparison based on model preference results across brain regions. An overview of BP_ND_ values estimated from each model (direct and indirect) can be found in Table 5.

**Table 5:** Regional binding potential (BP_ND_ or DVR–1) estimates from the different methods for the tracer dose group (*n* = 10). Data are expressed as mean ± SD

		DVR–1		BP_ND_		
Region	2TCM	1TCM	SA	FRTM	SRTM	SRTM-2C
Tracer dose						
Frontal cortex	2.09 ± 0.23	2.75 ± 0.36	1.94 ± 0.42	1.84 ± 0.20	1.92 ± 0.20	1.93 ± 0.20
Hippocampus	1.71 ± 0.21	2.28 ± 0.26	1.69 ± 0.32	1.53 ± 0.19	1.51 ± 0.19	1.59 ± 0.18
Cerebellum	0.75 ± 0.08	0.96 ± 0.14	0.77 ± 0.11	0.70 ± 0.07	0.68 ± 0.08	0.65 ± 0.08
Medulla	0.09 ± 0.03	0.12 ± 0.03	0.08 ± 0.03	0.08 ± 0.07	0.07 ± 0.04	0.05 ± 0.03

Compared to the other models, 1TCM DVR–1 showed the largest overall difference to 2TCM DVR–1 values (26.9%), with 95% limits of agreement reaching more than 40% for all regions (Table 4). Estimates of DVR–1 from SA demonstrated an overall small difference to 2TCM DVR–1 values (−4.21%) but with wide 95% limits of agreement (−39.8 to 31.37%), indicating an overall higher variability in parameter estimation (Fig. 6). All reference models (FRTM, SRTM, and SRTM-2C) underestimated BP_ND_ (differences of −13.9, −15.9, and −23.8%, respectively) in comparison with DVR–1 from the 2TCM.

Simulated data exhibited a similar pattern in the Bland-Altman plot (Fig. 7b). FRTM, SRTM, and SRTM-2C resulted in an underestimation of BP_ND_, with a (negative) bias (−0.75, −1.03, and −0.88%, respectively) that showed a slight increase for higher levels of binding (larger k_3_). SA displayed negligible bias (0.14%), independent of the binding level. DVR–1 from both the 1TCM and the 2TCM resulted in a positive bias, with 40.5% BP_ND_ overestimation for the 1TCM and a 5.6% overestimation for the 2TCM.

## Discussion

The 1TCM and the SRTM were previously established as methods of choice for the quantification of [^11^C]flumazenil in human studies [13]. Since a direct translation from the clinical to the pre-clinical setting, or vice-versa, is not recommendable, an assessment must be performed to determine whether the clinically used 1TCM and SRTM are also suited for the analysis of [^11^C]flumazenil in animal studies. Therefore, the present study evaluated different pharmacokinetic models for the quantification of [^11^C]flumazenil binding specifically for the rat brain.

The distribution pattern seen in the TD group was comparable to what was seen in human studies, as well as autoradiography studies of the rat brain [20, 30], displaying higher uptake in the cortical regions and the lowest in pons and medulla. However, it is interesting to note that the V_T_ in the pons was more than two times higher than what has been reported in human studies [13, 31]. In fact, both medulla and pons of the TD group exhibited a relatively high V_T_ (V_T_ > 2) for low-density regions, independently of the model used for quantification. Since these high V_T_ values could have been related to spillover effects from neighboring regions, the delineation of pons and medulla VOIs from the original atlas was limited in order to further separate the regions and minimize the potential spillover. Moreover, pre-saturation with cold flumazenil resulted in a clear decrease of the V_T_ in the pons of approximately 35%, as compared with the V_T_ values observed in the TD group. These results suggest the presence of a substantial level of specific binding in the rat pons, which is in line with a considerable displacement of a fluorinated version of flumazenil in that region, reported in a study by Dedeurwaerdere et al. [20]. For this reason, the pons cannot be considered a “true reference region” in rat studies, and the estimation of BP_ND_ from the reference tissue-based models are negatively biased.

In terms of model preference, regions with high density of GABA_A_ receptors (frontal cortex and hippocampus) followed the same behavior as what has been described in humans. These regions showed a strong (70%) preference for the 1TCM according to AIC and in accordance with the spectra of SA analysis. However, a closer look into the results suggests that the 2TCM resulted in similar AIC values (e.g., 143.7 ± 7.1 for the 1TCM against 144.8 ± 9.6 for the 2TCM in the frontal cortex), with both models providing similar V_T_ values. In fact, no statistical differences were found between 1TCM and 2TCM V_T_ values for those regions. Such findings are in line with what has previously been reported for human studies [11], where the authors state that expanding from a 1TCM to a 2TCM configuration did not improve the quality of the fit in high-density regions. These observations could be explained by the relative size between specific and non-specific compartments for these regions. In the presence of high levels of binding, the contribution of the non-specific component to the overall signal becomes too small, and the two compartments cannot be kinetically distinguished. Our results suggest that the 1TCM could be applied for these regions due to its reduced complexity compared to the 2TCM, despite the fact that both models provide similar AIC, V_T_, and BP_ND_ values. However, applying the same model to all regions of interest is advisable. In that way, potential individual or group differences in estimated parameters are not affected by differences in model choice and would therefore better represent any underlying physiological changes. Thus, the decision as to which of the two models (1TCM and 2TCM) should be applied must also incorporate the performance of these models for the low-density regions.

Indeed, one important finding of this study was the observation that the 1TCM is not suited for the quantification of low-density regions. The first indication is the preference displayed by these regions for the 2TCM, supported by AIC values, visual inspection of TAC fits (Fig. 3a, b), and kinetic spectra from SA. In fact, especially for the pons, the 1TCM was not preferred for any of the animals of the TD group. Moreover, V_T_ values were significantly different between models for low-binding regions, demonstrating that the various models cannot be used interchangeably in this case. Instead, the 2TCM provided the best fits, while the 1TCM consistently underestimated V_T_ compared to those from 2TCM. While the SA provided V_T_ values similar to those from 2TCM, the difference between the methods was still statistically significant and the standard deviation observed in this method was the largest of the three. This variability in the V_T_ estimates could be explained by the known sensitivity of this method to noise [25], but it could also be related to the wide definition of the basis function settings used in the study. Therefore, based on the present results, SA should be considered carefully for the analysis of rat data. It is interesting to notice, however, the intermediate behavior displayed by cerebellum in terms of model performance. In this region, although the model preference based on AIC strongly favored the 2TCM, differences between 1TCM or SA and 2TCM-derived V_T_ values were not so marked. This could be related to the intermediate levels of α subunits in this region [30].

Regarding the PS group, the analysis of model preference and performance produced results similar to those of the low-density regions, with lower AIC values for the 2TCM. These results were surprising, as the pre-saturation was expected to completely block the second (specific) compartment [32] and to leave only the first compartment intact (i.e., the compartment related to the free and non-displaceable components). Due to the similarity in model preference between PS group and low-density regions of the TD group, it was hypothesized that model preference is affected by the levels of specific binding present in a particular region. Therefore, simulated data were generated in order to substantiate this hypothesis, and the results obtained from the analysis seemed to support it. More specifically, both the 1TCM and the 2TCM showed good agreement to theoretical values for TACs with high V_T_, and the bias increased for TACs with lower V_T_. Moreover, the TACs with high binding and therefore high V_T_ values showed smaller differences in AIC between 1TCM and 2TCM fits, while this difference increased for lower V_T_ values. The differences in model preference and performance seen between the simulated and the animal datasets can be explained by the noiseless character of the generated TACs.

As previously mentioned, the rat pons demonstrated a considerable level of specific binding. Coupled with the underestimation in V_T_ seen from the 1TCM, these results could explain the poor performance of reference models. First, the specific binding seen in the pons results in an underestimation of BP_ND_ by direct estimation based on reference models. Second, the fact that the pons is better described by a 2TCM directly violates one of the assumptions used in the FRTM and SRTM, where the reference region should be adequately described by a 1TCM configuration [27]. Our results confirmed the violation of this assumption, and both SRTM and FRTM were affected in a similar way, displaying similar differences in BP_ND_ estimates as compared to DVR–1 from the 2TCM. Such an effect has larger impact for high-binding regions and results in considerable underestimation of BP_ND_ in absolute terms [33]. In order to allow a different configuration for the reference tissue, the SRTM-2C was also tested. Interestingly, the SRTM-2C showed a small improvement compared to the other reference models for high-binding regions (Table 3). Indeed, fits of those regions showed lower AIC values for the SRTM-2C compared to the SRTM (data not shown). However, the 2TCM-2C resulted in larger differences to 2TCM DVR–1 for cerebellum and medulla, which could be due to a combination between low amounts of specific binding and the larger number of model parameters, resulting in increased uncertainty in parameter estimation. In that context, SRTM represented the most robust reference model for the animal data. Yet, it should be mentioned that both plasma input-derived DVR–1 and SRTM-based BP_ND_ estimates will be negatively biased by the presence of specific binding in the reference region (pons), as discussed below. Moreover, it is also important to notice that although radiometabolites have the potential to introduce bias in estimation of model parameters, the reported major metabolites of [^11^C]flumazenil are polar compounds and, as such, are expected not to enter the brain [34].

In summary, the quantification of [^11^C]flumazenil binding in rat studies should not be performed with the 1TCM, as it is the case in human studies. In fact, the 2TCM is required for an accurate V_T_ estimation in low-density regions such as the pons, and it provides V_T_ values equivalent to 1TCM for the high-density regions. In the case of reference-based modeling, the use of the pons as a reference region requires careful consideration. Since the rat pons demonstrated a considerable level of specific binding, any direct estimation of BP_ND_ via reference models will suffer from underestimation of tracer binding. However, the pons might be considered a pseudo-reference region [35], provided the experimental conditions and physiological state of the animals does not affect tracer delivery and binding in this region across the different groups. Such a condition might not be met for different study designs. In those cases, a first validation using plasma input kinetic analysis in a subsample of the data is required in order to ensure that pons could be used as pseudo-reference region. Once the pons has been validated as a pseudo-reference region, reference-based modeling could be useful in providing a simple and robust alternative for BP_ND_ estimation without arterial sampling. In that case, SRTM would be preferable due to its robust implementation, smaller number of fit parameters, and good agreement to DVR–1 from the 2TCM.

## Conclusions

This study showed that [^11^C]flumazenil kinetics is different between rats and humans, and indicate that kinetic models used in clinical studies are not necessarily suited in the pre-clinical setting. For high-density regions, both 1TCM and 2TCM estimated comparable V_T_ values and resulted in similar quality of fits (AIC). However, the application of the 2TCM in low-density regions resulted in higher quality of fits, both from visual inspection and AIC values. Moreover, the clinically applied 1TCM was not able to satisfactorily fit the data (visual inspection) of those regions, nor in the case of pre-saturation with cold flumazenil. Therefore, the 2TCM is the preferred plasma input model for the quantification of [^11^C]flumazenil in the rat brain. Among the various reference tissue models tested, SRTM was preferred providing BP_ND_ estimates in good agreement with plasma input 2TCM based DVR–1 values.

## References

[1] Odano I, Halldin C, Karlsson P, Varrone A, Airaksinen AJ, Krasikova RN, et al. [18 F]Flumazenil binding to central benzodiazepine receptor studies by PET. Neuroimage [Internet]. Elsevier Inc.; 2009. 45(3):891–902. Available from: http://dx.doi.org/10.1016/j.neuroimage.2008.12.005 10.1016/j.neuroimage.2008.12.005 19136064

[2] Heiss WD, Grond M, Thiel A, Ghaemi M, Sobesky J, Rudolf J (1998). Permanent cortical damage detected by flumazenil positron emission tomography in acute stroke. Stroke.

[3] Heiss WD, Kracht L, Grond M, Rudolf J, Bauer B, Wienhard K (2000). Early [(11)C]Flumazenil/H(2)O positron emission tomography predicts irreversible ischemic cortical damage in stroke patients receiving acute thrombolytic therapy. Stroke [Internet].

[4] Atack JR, Scott-stevens P, Beech JS, Fryer TD, Hughes JL, Cleij MC (2007). Comparison of lorazepam [7-chloro-5-(2-chlorophenyl)-1,3-dihydro-3-hydroxy-2H-1, 4-benzodiazepin-2-one] occupancy of rat brain gamma—aminobutyric acid a receptors measured using in vivo [3H]flumazenil (8-fluoro 5,6-dihydro-5-methyl-6-oxo-4H-imidaxzo[1. J Pharmacol Exp Ther.

[5] Geeraerts T, Coles JP, Aigbirhio FI, Pickard JD, Menon DK, Fryer TD (2011). Validation of reference tissue modelling for [11C]flumazenil positron emission tomography following head injury. Ann Nucl Med [Internet].

[6] Lamusuo S, Pitkänen A, Jutila L, Ylinen A, Partanen K, Kälviäinen R (2000). [11 C]Flumazenil binding in the medial temporal lobe in patients with temporal lobe epilepsy: correlation with hippocampal MR volumetry, T2 relaxometry, and neuropathology. Neurology [Internet].

[7] Heiss WD, Sobesky J, Smekal UV, Kracht LW, Lehnhardt FG, Thiel A (2004). Probability of cortical infarction predicted by flumazenil binding and diffusion-weighted imaging signal intensity: a comparative positron emission tomography/magnetic resonance imaging study in early ischemic stroke. Stroke.

[8] Pascual B, Prieto E, Arbizu J, Marti-Climent JM, Peñuelas I, Quincoces G (2012). Decreased carbon-11-flumazenil binding in early Alzheimer’s disease. Brain [Internet].

[9] Gunn RN, Gunn SR, Cunningham VJ (2001). Positron emission tomography compartmental models. J Cereb Blood Flow Metab [Internet].

[10] Millet P, Graf C, Buck A, Walder B, Ibáñez V (2002). Evaluation of the reference tissue models for PET and SPECT benzodiazepine binding parameters. Neuroimage.

[11] Koeppe RA, Holthoff VA, Frey KA, Kilbourn MR, Kuhl DE (1991). Compartmental analysis of [11C]flumazenil kinetics for the estimation of ligand transport rate and receptor distribution using positron emission tomography. J Cereb Blood Flow Metab [Internet].

[12] Price JC, Mayberg HS, Dannals RF, Wilson AA, Ravert HT, Sadzot B (1993). Measurement of benzodiazepine receptor number and affinity in humans using tracer kinetic modeling, positron emission tomography, and [11C]flumazenil. J Cereb Blood Flow Metab.

[13] Klumpers UM, Veltman DJ, Boellaard R, Comans EF, Zuketto C, Yaqub M (2008). Comparison of plasma input and reference tissue models for analysing [11C]flumazenil studies. J Cereb Blood Flow Metab [Internet]..

[14] Halldin C, Farde L, Litton J, Hall H, Sedvall G (1992). [11C]Ro 15-4513, a ligand for visualization of benzodiazepine receptor binding. Psychopharmacology (Berl) [Internet].

[15] Hoekzema E, Rojas S, Herance R, Pareto D, Abad S, Jiménez X, et al. In vivo molecular imaging of the GABA/benzodiazepine receptor complex in the aged rat brain. Neurobiol Aging [Internet]. Elsevier Inc.; 2012; 33(7):1457–65. Available from: http://dx.doi.org/10.1016/j.neurobiolaging.2010.12.006 10.1016/j.neurobiolaging.2010.12.006 21272959

[16] Rojas S, Martín A, Pareto D, Herance JR, Abad S, Ruíz A, et al. Positron emission tomography with 11C-flumazenil in the rat shows preservation of binding sites during the acute phase after 2 h-transient focal ischemia. Neuroscience [Internet]. Elsevier Inc.; 2011 May 19;182:208–16. Available from: http://dx.doi.org/10.1016/j.neuroscience.2011.03.013 10.1016/j.neuroscience.2011.03.013 21402129

[17] Parente A, Feltes PK, Vallez Garcia D, Sijbesma JWA, Moriguchi Jeckel CM, Dierckx RAJO (2016). Pharmacokinetic analysis of 11C-PBR28 in the rat model of herpes encephalitis: comparison with (R)-11C-PK11195. J Nucl Med.

[18] Vállez García D, de Vries EFJ, Toyohara J, Ishiwata K, Hatano K, Dierckx RAJO (2015). Evaluation of [(11)C]CB184 for imaging and quantification of TSPO overexpression in a rat model of herpes encephalitis. Eur J Nucl Med Mol Imaging.

[19] de Vries EFJ, Dierckx RA, Klein HC (2006). Nuclear imaging of inflammation in neurologic and psychiatric disorders. Curr Clin Pharmacol [Internet].

[20] Dedeurwaerdere S, Gregoire M-C, Vivash L, Roselt P, Binns D, Fookes C (2009). In-vivo imaging characteristics of two fluorinated flumazenil radiotracers in the rat. Eur J Nucl Med Mol Imaging [Internet].

[21] Någren K, Halldin C (1998). Methylation of amide and thiol functions with [11C]methyl triflate, as exemplified by [11C]NMSP[11C]flumazenil and [11C]methionine. J Label Compd Radiopharm.

[22] Hudson HM, Larkin RS (1994). Accelerated image reconstruction using ordered subsets of projection data. IEEE Trans Med Imaging [Internet].

[23] Vállez Garcia D, Casteels C, Schwarz AJ, Dierckx RAJO, Koole M, Doorduin J (2015). A standardized method for the construction of tracer specific PET and SPECT rat brain templates: validation and implementation of a toolbox. PLoS One [Internet].

[24] Cunningham VJ, Jones T (1993). Spectral analysis of dynamic PET studies. J Cereb Blood Flow Metab [Internet].

[25] Turkheimer F, Sokoloff L, Bertoldo A, Lucignani G, Reivich M, Jaggi JL (1998). Estimation of component and parameter distributions in spectral analysis. J Cereb Blood Flow Metab [Internet].

[26] Veronese M, Rizzo G, Bertoldo A, Turkheimer FE (2016). Spectral analysis of dynamic PET studies: a review of 20 years of method developments and applications. Comput Math Methods Med [Internet].

[27] Lammertsma AA, Hume SP (1996). Simplified reference tissue model for PET receptor studies. Neuroimage [Internet]..

[28] Innis RB, Cunningham VJ, Delforge J, Fujita M, Gjedde A, Gunn RN (2007). Consensus nomenclature for in vivo imaging of reversibly binding radioligands. J Cereb Blood Flow Metab [Internet].

[29] Akaike H, Parzen E, Tanabe K, Kitagawa G (1998). A new look at the statistical model identification. Selected Papers of Hirotugu Akaike [Internet].

[30] Wisden W, Laurie DJ, Monyer H, Seeburg PH (1992). The distribution of 13 GABAA receptor subunit mRNAs in the rat brain. I. Telencephalon, diencephalon, mesencephalon. J Neurosci [Internet].

[31] Miederer I, Ziegler SI, Liedtke C, Spilker ME, Miederer M, Sprenger T (2009). Kinetic modelling of [11C]flumazenil using data-driven methods. Eur J Nucl Med Mol Imaging.

[32] Litton JE, Hall H, Pauli S (1994). Saturation analysis in PET—analysis of errors due to imperfect reference regions. J Cereb Blood Flow Metab..

[33] Salinas C A, Searle GE, Gunn RN. The simplified reference tissue model: model assumption violations and their impact on binding potential. J Cereb Blood Flow Metab [Internet]. Nature Publishing Group; 2015;35(2): 304–11. Available from: http://www.nature.com/doifinder/10.1038/jcbfm.2014.202 10.1038/jcbfm.2014.202 PMC4426748 25425078

[34] Amini N, Nakao R, Schou M, Halldin C (2013). Identification of PET radiometabolites by cytochrome P450, UHPLC/Q-ToF-MS and fast radio-LC: applied to the PET radioligands [11C]flumazenil, [18 F]FE-PE2I, and [11C]PBR28. Anal Bioanal Chem.

[35] Lyoo CH, Ikawa M, Liow J-S, Zoghbi SS, Morse C, Pike VW, et al. Cerebellum can serve as a pseudo-reference region in Alzheimer’s disease to detect neuroinflammation measured with PET radioligand binding to translocator protein (TSPO). J Nucl Med [Internet]. 2015; 701–7. Available from: http://jnm.snmjournals.org/cgi/doi/10.2967/jnumed.114.146027 10.2967/jnumed.114.146027 PMC4839390 25766898

